# Mitochondrial Genome and Phylogenetic Analysis of the Narrownose Smooth-Hound Shark *Mustelus schmitti* Springer, 1939

**DOI:** 10.3390/ani14233396

**Published:** 2024-11-25

**Authors:** Walter Nisa-Castro-Neto, Paulo Guilherme Carniel Wagner, Diéssy Kipper, Vinicius Proença da Silveira, André Salvador Kazantzi Fonseca, Nilo Ikuta, Vagner Ricardo Lunge

**Affiliations:** 1 Organização para a Pesquisa e a Conservação de Esqualos no Brasil (PRÓ-SQUALUS), Torres 905560-000, RS, Brazil; nisacn@prosqualus.org (W.N.-C.-N.); paulo.wagner@ibama.gov.br (P.G.C.W.); 2 Instituto de Biotecnologia/Programa de Pós-Graduação em Biotecnologia (PPGBIO), Universidade de Caxias do Sul (UCS), Caxias do Sul 95070-560, RS, Brazil; 3 Instituto Brasileiro do Meio Ambiente e dos Recursos Naturais Renováveis (IBAMA/RS)/Centro de Triagem de Animais Silvestres (CETAS/RS), Porto Alegre 90160-070, RS, Brazil; 4 Simbios Biotecnologia, Cachoeirinha 94950-000, RS, Brazil; diessykipper@hotmail.com (D.K.); viniciusdasilveir@gmail.com (V.P.d.S.); fonseca@simbios.com.br (A.S.K.F.); ikuta@simbios.com.br (N.I.)

**Keywords:** complete mtDNA, comparative mitogenomics, elasmobranch phylogenetics, philogenomics, Triakidae, *Mustelus schmitti*, houndsharks, threat species, south Brazil, western south Atlantic

## Abstract

Southern Brazil is home to a large biodiversity of elasmobranchs from the Brazilian coast. Several genera and species of small sharks of the Triakidae family live in this marine environment. Studies on these shark species are scarce, with few genetic data and little information on animal population structures. The present study aimed to sequence the complete mitochondrial genome (mtDNA) of the endangered species *Mustelus schmitti* (narrownose smooth-hound shark) and to perform a phylogenetic analysis of the Triakidae family. The mtDNA sequenced here was 16,764 bp long and possessed the usual 13 mitochondrial protein coding genes (PCGs), 22 tRNAs, two rRNAs (12S and 16S) and a large *D-loop* DNA sequence, presenting an overall organization similar to other species from the genus *Mustelus*. Phylogenetic analyses were performed using a dataset containing this new mtDNA and 59 other mitochondrial genomes of the Carcharhiniformes species (including 14 from the Triakidae family), using the Maximum Likelihood (ML) method. All the species of the Triakidae family were clustered into a monophyletic topology group. In addition, polyphyly was observed in *Galeorhinus galeus*, *Hemiatrakis japanica*, *Triakis megalopterus* and *Triakis semifasciata*. In conclusion, this study contributes to a deeper understanding of the genetic diversity of sharks and represents an important step towards the conservation of these endangered animals.

## Simple Summary

The Southwest Atlantic Ocean (SAO) is a biodiversity hotspot for elasmobranchs. This marine subclass of animals is deeply threatened by anthropogenic activities and many species are at imminent risk of extinction, such as sharks of the genus *Mustelus*. Scientific reports on the biology, ecology and genetic identity of the narrownose (*Mustelus schmitti*), the smalleye (*Mustelus higmani*) and the striped (*Mustelus fasciatus*) smooth-hound sharks from the family Triakidae are few in the literature. This study describes the sequencing of the first complete mitochondrial genome of a Chondrichthyes from the SAO coast (*Mustelus schmitti*, a shark classified as critically endangered by the International Union for Conservation of Nature—IUCN) and presents the phylogenetic analysis of this species. The scientific knowledge obtained here will help in elasmobranch conservation programs, in future studies on population ecology and genetics and in fisheries control for this and other small coastal shark species at risk in SAO.

## 1. Introduction

Chondrichthyes are a group of vertebrates with an ancestral evolutionary history dating back 400 million years [1,2]. The order Carcharhiniformes is the largest, most diverse and widely distributed taxon of sharks in the world’s seas and oceans, containing 10 families and approximately 291 species [3]. The genus *Mustelus* of the family Triakidae includes 28 species of small demersal sharks found in temperate and tropical waters of the continental shelves of all oceans [4]. In the Southwest Atlantic Ocean (SAO), animals of this genus can be found from Rio de Janeiro, Brazil, to Patagonia, Argentina [5]. Five species of *Mustelus* are frequently observed on the Brazilian coast: *Mustelus canis*, *Mustelus fasciatus*, *Mustelus higmani*, *Mustelus norrisi* and *Mustelus schmitti* [6]. These shark species are under varying levels of threat along the entire Brazilian coast [7,8].

In southern Brazil, fishing for large sharks was quite intensive in the 1980s [9]. As a consequence, populations of these animals declined in the 1990s and fishing became economically unviable, mainly for the more traditional fisheries. Therefore, fishermen directed their efforts to smaller and more coastal shark species, such as *Squatina* spp., *Squalus* spp., *Galeorhinus galeus*, *Rhizoprionodon* spp., *Carcharhinus* spp., *Sphyrna* spp. and the three main species of the genus *Mustellus* (*M. canis*, *M. fasciatus* and *M. schimitti*) [9,10]. The large reduction in populations of several shark species in the SAO resulted in conservation efforts for these ecologically important and vulnerable animals in Brazil [11,12]. Sharks were classified into risk of extinction and/or legal protection categories in 2004 (Normative Instruction 005/2004, Brazil). Two *Mustelus* species (*M. fasciatus* and *M. schmitti*) are sharks with small distribution and endemic to SAO [13,14]. They should be protected to avoid a collapse in shark populations, as observed in other oceans [15,16,17,18,19,20,21].

The narrownose smooth-hound shark (*M. schmitti*) is a coastal species (ICMBio, Brazil and IUCN) living in the seas from southern Brazil to Argentina [22,23,24]. The reproductive cycle is annual, and gestation takes between 11 and 12 months [25]. *M. schmitti* has slow growth and low fecundity, limiting recruitment and increasing vulnerability to overfishing. Catches in southern Brazil occur mainly in the winter and, in Uruguay, during summer and fall [26]. There is segregation of sexes and life stages [25,27]. Other information about behavior and reproduction was described previously [28].

Recent scientific articles have reported the sharks’ population structures [29,30,31]. Some studies emphasize the importance of sequencing genes and genomes to understand the population structure of many shark species [32,33,34,35]. The evolutionary history of chondrichthyans is also being elucidated with genetic data [1,36,37,38,39]. Most phylogenetic reports are based on a restricted number of nuclear and/or mitochondrial genes [37,40,41]. Next-generation sequencing (NGS) techniques have enabled reliable and accurate analysis of mitochondrial genomes (mtDNA) in recent years [42,43]. mtDNA data can be especially useful for identifying chondrichthyans, as well as for elucidating the occurrence of population units and understanding migratory flows [44,45]. All this genetic information is demonstrating more clearly the phylogeny of Chondrichthyes, as well as providing precise insights into the evolutionary relationships of these animals [46,47].

The present study sequenced the mitochondrial genome of the narrownose smooth-hound shark *M. schmitti* and performed a more complete phylogenetic analysis of the family Triakidae. The results reported here will help to elucidate the evolution of this endangered shark species and its relationship with other chondrichthyans.

## 2. Materials and Methods

### 2.1. Specimen Collection

**Figure 1:**
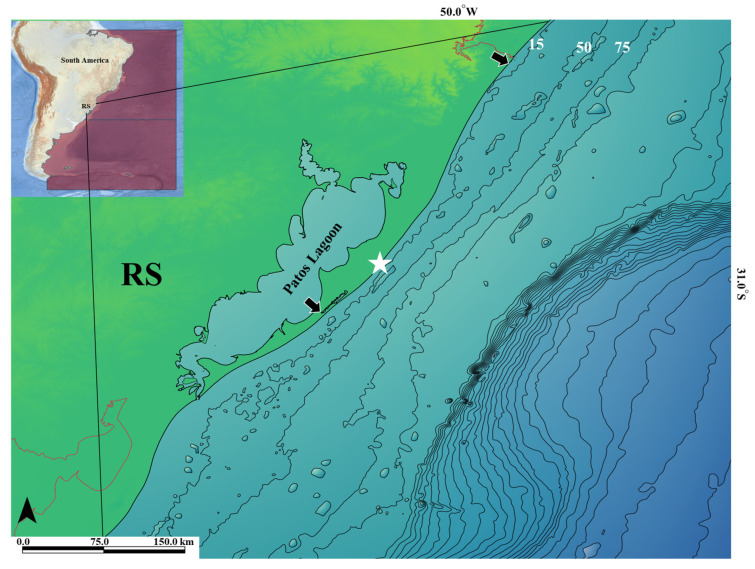
Sampling place of the *Mustelus schmitti* specimen (white star) on the coast of Rio Grande do Sul (RS) state, south Brazil. Coastal isobaths of 15 to 75 m are shown. The Southwest Atlantic (SAO) can be seen in purple in the top left corner. The arrows mark the northern and southern limits of the sampling in this research study approval by ICMBio/SISBIO.

One juvenile specimen of *M. schmitti* was collected from Praia de Mostardas, Mostardas, Rio Grande do Sul, Brazil (-30.864777 S/-50.595253 W) on 30th May 2023. The catch was accidental, using a coastal gillnet, positioned about 70 m from the beach line perpendicular to the shore. The net had been in place since 7:00 AM and was removed at 4:00 PM (Figure 1). Ethical procedures were adopted according international guidelines [48] and permission to perform this research was obtained from ICMBio/SISBIO (no. 71583) and SISGEN (no. ACA1D5C).

### 2.2. DNA Extraction, Sequencing and mtDNA Assembly

Genomic DNA was extracted using the PureLink^TM^ Genomic DNA Mini Kit according to the manufacturer’s instructions. Genome sequencing of *M. schmitti* was performed by Neoprospecta (Florianópolis, Brazil) using the MiSeq platform and a 305/205 paired-end library approach and prepared using the Illumina DNA Prep Kit. Trimmomatic v. 0.33 [49] was used to trim raw sequence reads and remove low-quality bases. The quality of trimmed reads was assessed using FastQC v. 0.11.2 (http://www.bioinformatics.babraham.ac.uk/projects/fastqc/, accessed on 31 June 2024). The reads were mapped against a reference mitogenome from a closely related species in Geneious Prime^®^ v. 2024.0.5 (Biomatters, Auckland, New Zealand, www.geneious.com). The reference mtDNAs are presented in Table 1. Subsequently, the mapped Illumina reads were assembled de novo using SPAdes v. 3.6.0 [50]. The quality of draft genomes was evaluated using QUAST v. 4.0 [51]. As a final step, the total DNA-seq reads and assembly were mapped to the mtDNA from a closely related species in Geneious Prime software to confirm the quality of the assembly, correct potential assembly gaps and finally close the circular molecule.

### 2.3. Annotation and Sequence Analysis

The complete *M. schmitti* mtDNA was annotated using MitoAnnotator on the MitoFish website (http://mitofish.aori.u-tokyo.ac.jp/annotation/input.html (accessed on 31 June 2024) [52]. The programs RNAmmer (https://services.healthtech.dtu.dk/services/RNAmmer-1.2/ (accessed on 31 June 2024)) [53] and tRNA scan-SE (http://lowelab.ucsc.edu/tRNAscan-SE/ (accessed on 31 June 2024)) [54] were used to confirm the ribosomal RNA (rRNA) and the transfer RNA (tRNA) annotation results, respectively. The boundaries of the protein-coding genes (PCGs), rRNA genes and tRNA genes were refined manually by comparison with the annotated elasmobranch mtDNAs from GenBank. The complete mitochondrial genome obtained here was deposited in GenBank under the accession number PQ182775.

The assembled genome of *M. schmitti* was initially annotated using the MitoAnnotator and the nucleotide composition and genetic distance of the entire mitochondrial genome were analyzed using the MEGA11 v. 11.0.6 software [55].

### 2.4. Phylogenetic Analyses

A total of 59 complete mtDNAs of Carcharhiniformes species (14 Triakidae) were obtained from NCBI [45] and used to construct the dataset (Table 1). *Chiloscyllium griseum* (NC_017882), from the family Hemiscylliidae, and *Lamna ditropis* (NC_024269), from the family Lamnidae, were used as outgroups. The complete sequences (including D-loops) were aligned using the CLUSTAL W algorithm [56] plugin in the Geneious Prime^®^ v. 2024.0.5. The phylogenetic relationships were reconstructed with the Maximum Likelihood (ML) method implemented in W-IQ-TREE web server [57], the optimal nucleotide substitution model selected using ModelFinder (GTR + F + I + G4) [58] and 1000 replicates of the ultrafast bootstrap approximation [59]. In addition, a Bayesian Inference (BI) phylogenetic tree was constructed in Geneious Prime^®^ v. 2024.0.5, under 2 parallel runs and 2,000,000 generations. The initial 10% of sampled data was discarded as burn-in with default settings. FigTree v. 1.4.4 (http://tree.bio.ed.ac.uk/software/figtree/, accessed on 31 June 2024) was used to annotate the resulting phylogeny.

## 3. Results

### 3.1. Genome Structure, Composition and Asymmetry

**Figure 2:**
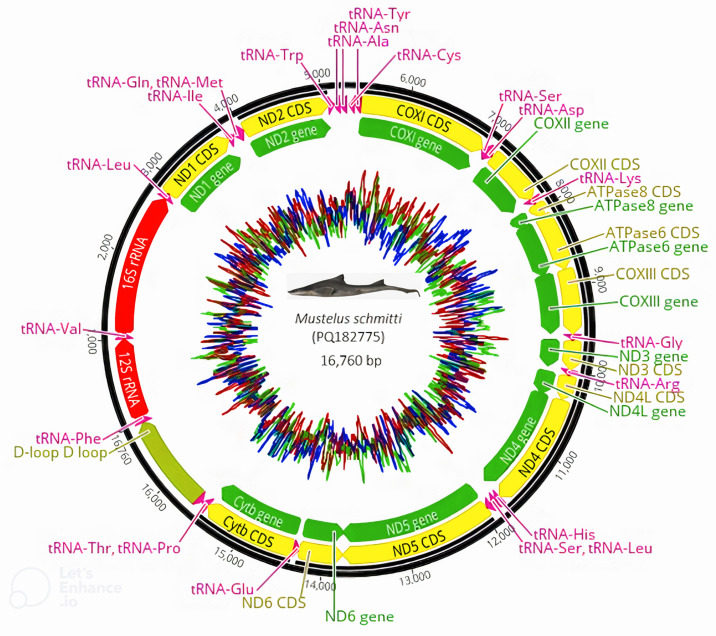
Circular mitochondrial genome map of the *M. schmitti*. Genomic map constructed with Geneious Prime software. Colors denote type annotations (Yellow: CDS; green: genes; red: rRNA; pink: tRNA). The content of ACTG is represented on the inside (red:A; blue:C; green:T; yellow:G).

The complete mtDNA of *M. schmitti* is 16,764 base pairs (bp) long, which is about the expected size for a shark genus. It also contains the typical genes, including 13 PCGs, 22 tRNAs, two rRNAs (12S and 16S) and a large D-loop. The gene order and the gene transcription directions in the complete *M. schmitti* mtDNA are consistent with those demonstrated for most vertebrate mtDNAs (Figure 2 and Table 2).

The overall percentages of the base composition are 30.7% A, 24.5% C, 14% G and 30.7% T. In a gene-by-gene comparison, the AT content is greater than the GC content in most genes. The AT ranged from 57% (tRNA-Thr) to 79.7% (tRNA-His), while the GC ranged from 30% (tRNA-Gly) to 56.5% (tRNA-Pro) (Table 2).

### 3.2. Protein Coding Regions, Transfer RNA and Ribosomal RNA

The 13 PCGs totaled 11,429 bp in length, accounting for 68.2% of the mtDNA and encoding a total of 3800 amino acids. All the PCGs are encoded by the heavy strand (H-strand), except for the *ND6* gene, encoded by the light strand (L-strand). The length of the PCGs ranges from 168 bp (*ATPase8*) to 1830 bp (*ND5*) (Table 2). All the PCGs started with an ATG codon, except the *COXI* (GTG) and *ND6* (CTA) genes, and finished with the three traditional stop codons (TAA, TAG, TGA), except *ND6*, which was terminated by a rare CAT codon (Figure 2, Table 2).

The 22 tRNA genes ranged from 67 bp (tRNA-*Ser2*) to 75 bp (tRNA*Leu1*) in length and were located between rRNAs and PCGs. The total length of the tRNAs genes is 1553 bp, accounting for 9.3% of the whole mtDNA. Fourteen tRNAs are encoded on the H-strand and the remaining tRNAs are encoded on the L-strand. All tRNAs have a high AT content (79.7%) (Table 2).

The 12S and 16S rRNA genes are 953 bp and 1670 bp long, respectively. They are located between tRNA-*Phe* and tRNA-*Leu*, and separated by tRNA-*Val*, similar to most fish mtDNAs. All rRNAs have a high AT content (62.5%) (Table 2).

**Figure 3:**
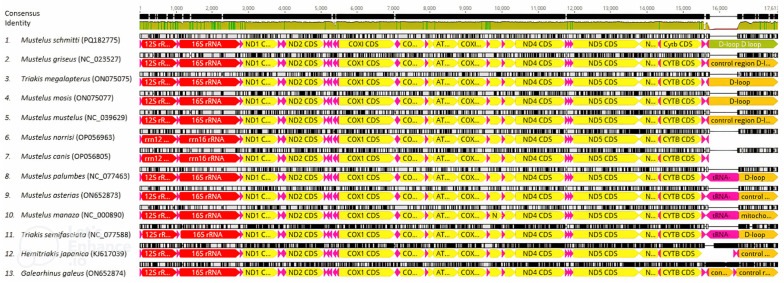
Alignment of the Triakidae mtDNAs showing all *Mustelus* species. Colors denote type annotations (Yellow: CDS; red: rRNA; pink: tRNA; orange: D-loops).

An additional analysis was performed within the 13 shark species from the family Triakidae. All the Triakidae mtDNAs contained the same 13 PCGs: ND1, ND2, COX1, COX2, ATP8, ATP6, COX3, ND3, ND4L, ND4, ND5, ND6 and Cytb. ND5 was the longest (1830 bp), while the ATP8 was the shortest (168 bp). These mtDNAs also had a small rRNA subunit (12S) and another large rRNA subunit (16S), ranging from 951 to 954 bp and 1648 to 1672 bp in length, respectively. Twelve mtDNAs had 22 tRNA genes, but *Galeorhinus galeus* (GenBank access ON652874) presented 23 genes (the tRNA-*Thr* gene is duplicated). The differences in the mtDNA sizes among species from Triakidae family are due to length of the control regions (D-loops). The D-loops ranged from 974 bp in *Triakis semifasciata* (GenBank access NC077588) to 1.786 bp in *G. galeus* (GenBank access ON652874) (Figure 3).

### 3.3. Phylogenetic Analysis

**Figure 4:**
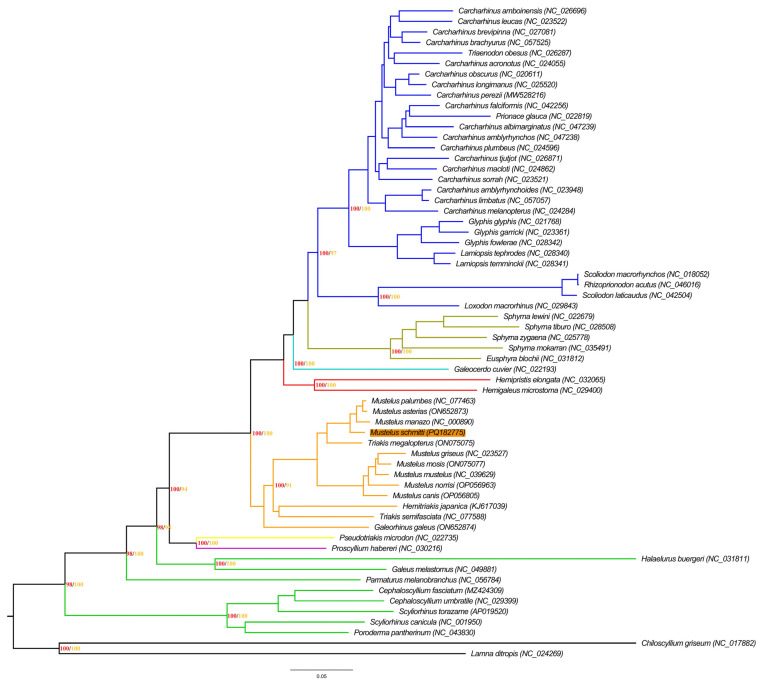
The phylogenetic tree of the Carcharhiniformes species. The orange square indicates the sequence species generated in this study (*Mustelus schmitti*). Bootstrap support (right = yellow) and Bayesian posterior probability (left = red) values of each main clade are displayed next to the nodes. Clades are colored according to families: blue = Carcharhinidae; sepia = Sphyrnidae; light blue = Galeocerdidae; red = Hemigaleidae; orange = Triakidae; yellow = Proscylliidae; purple = Pseudotriakidae; green = Scyliorhinidae. *Chiloscyllium griseum* and *Lamna ditropis* were used as outgroups.

The phylogenetic tree included 60 species from the seven main families of ground sharks (Carcharhiniformes): Carcharhinidae, Hemigaleidae, Proscylliidae, Pseudotriakidae, Scyliorhinidae, Sphyrnidae and Triakidae. The topology of the ML tree was consistent, presenting well-supported clades with high bootstrap probabilities (BPs). In addition, all the species from same families clustered together as expected (Figure 4).

Specifically, the family Triakidae presented *G. galeu* separately in a basal branch. The remaining twelve species clustered into three well separated branches. The *M. schmitti* mtDNA sequenced here clustered within four other species: *M. palumbes*, *M. asterias*, *M. manazo* and *T. megalopterus*. The remaining five Mustelus species (*M. griseus*, *M. mosis*, *M. mustelus*, *M. norrisi* and *M. canis*) clustered together in another branch, while *Hemitriakis japanica* and *Triakis semifasciata* clustered in a third one. Therefore, sister groups were observed within the members of the genus Mustelus (Figure 4).

The phylogenetic tree also demonstrated the paraphyly of the genera *Mustelus* and *Triakis*, since *T. megalopterus* occupied an initial branching position. *T. semifasciata*, which was also included in the phylogenetic analysis and was established as a sister group of the *M. schmitti* clade containing *M. manazo*, *M. asterias* and *M. palumbis*.

## 4. Discussion

The use of molecular genetic methods to characterize new marine animal species (including sharks) has increased substantially in the last decades [60]. Scientific studies identified and classified many new species, as well as demonstrating the genetic diversities and their gene flows [42,44,45]. It is necessary to study each species separately to reduce the risk of these animals’ extinction [61,62]. However, only ≅10% of the elasmobranchs were well-studied for genetic identity and population structures.

In the present study, the complete mtDNA from a *M. schmitti* specimen was originally sequenced (GenBank Accession PQ182775). Regarding general characteristics, *M. schmitti*’s mtDNA length and overall organization are similar to those of other sharks from the same family (Triakidae). The complete mtDNA of *M. schmitti* is 16,764 bp, the expected size for shark genomes (ranging from 16,677 bp in *C. falciformis* to 19,100 bp in *H. buergeri*) and in agreement with the mtDNA conservancy among elasmobranchs [43]. In addition, the overall genomic organization, with the presence of 13 PCGs, 22 tRNAs, two rRNAs and a large D-loop region, is typical of other vertebrates [63,64]. The overall nucleotide composition is also consistent with other studies and suggests a conservative trend throughout chondrichthyan evolution [65,66,67,68,69].

A more detailed comparative analysis within the family Triakidae demonstrated that the mtDNAs lengths and compositions of the different species were almost the same as those previously reported [43,45,70]. The base composition of *M. schmitti* differs from those of *M. canis* and *M. norrisi*, suggesting paraphilia of these triakid species, even though they occur in synchronous areas of the South Atlantic [71,72]. The conservation of the length and composition of the mtDNA from this family suggests evolutionary stability due to the strong selective pressure maintaining the mitochondrial gene order and sequences among chondrichthyans [73,74]. This highly conserved gene organization of *M. schmitti* PCGs in the mtDNA also highlights the similarity with other members of the same taxonomic order [75,76]. Notably, the presence of 13 PCGs is a pattern observed in many other shark species [77], with H-strand coding for most proteins, except for ND6 (coded L-strand) [66,72]. The variation in the length of the PCGs, from 168 bp (ATPase8) to 1830 bp (ND5), is also in line with other studies [78,79]. All this distribution has already been hypothesized to help in mitochondrial gene expression [80,81]. Finally, 12S and 16S rRNA genes are inserted between tRNA-*Phe* and tRNA-*Leu*, similarly to what has been observed in many elasmobranch mtDNAs [82]. The organization of rRNAs is fundamental for regulating gene expression and maintaining mitochondrial functionality [61].

The phylogenetic tree for Charchariniforms (updated with *M. schmitti*) provided a more comprehensive view of the relationships within the family Triakidae. First, all the genera and species clustered into a unique clade with 100% BPs, highlighting the taxonomic robustness of this family [66,83]. Second, the position of *G. galeus* in the basal branch of the Triakidae clade suggests a distant relationship with the other *Mustelus* species, consistent with the broad distribution patterns of this species across all oceans [42,70]. This polyphyletic arrangement is further supported by the vicariant processes and gene flows that have occurred throughout the taxon’s evolution [32,84,85]. However, the presence of polyphyletic arrangements among genera within the Triakidae clade reveals some unsolved questions [43,44,45]. Furthermore, the occurrence of sister groups among *Mustelus* spp. highlights the genetic proximity and overlap of the areas where these species occur in the oceans [36]. These results corroborated the importance of studying the geographical distributions of the elasmobranchs [45]. The evidence of paraphyly in the genera *Mustelus* and *Triakis*, with *T. megalopterus* occupying a basal position and grouping as a well-supported sister clade with the other *Mustelus* species, also suggests that these genera have undergone distinct and complex evolutionary processes. The separation of *T. semifasciata* as a sister group to the clade of *M. schmitti*, *M. manazo*, *M. asterias* and *M. palumbis*, with strong support, may also indicate historical events and fixation in coastal areas, and with limitations to single regions [66,78].

In a wider view of the phylogenetic tree, the clusters for Carcharhinidae and Triakidae exhibit interesting intra-familial relationships, suggesting some lineages may have undergone recent rapid radiations [42,86,87]. The difficulties in resolving these relationships are due to an incomplete sorting of the lineages, since not enough time has passed to reliably resolve some of these knots, because the permanent genetic variation of the ancestral species inherited by the immediate descendant species was not separated before speciation [88,89]. In this context, excess partitioning can reduce the number of informative sites needed to accurately group these taxa [90,91,92,93]. These findings highlight the need for more studies integrating phylogenetic, ecological and geographic data for a comprehensive understanding of evolution and diversification within the Triakidae [94,95].

It is also important to analyze the vicariance/gene flow processes that occurred throughout the evolution of Charchariniformes, especially for hound sharks [96,97,98]. *M. canis* and *M. norrisi* are found in the same geographic areas of the western Atlantic coast, *M. mosis* occurs on the eastern coast of the Indian Ocean and *M. griseus* in the western Pacific Ocean. *M. mosis* is distributed from the southern Indian Ocean along the western coast of Africa to the Mediterranean. They grouped into a single clade, highlighting the wide distribution of their ancestral species by the continental distribution and circulation of the Atlantic Ocean during the Turonian Period (93.9 million and 89.8 million years ago Ma). In contrast, the other two species of this family (*H. japanica* and *T. semifasciata*) grouped into another clade. These species now live in the Pacific Ocean and probably originated in this geographic area in the Turonian Period [99,100].

Regarding reproductive mode, *M. schmitti* fits into the same group as the species that exhibit aplacental viviparity [5]. This result is consistent with previous studies suggesting that shared reproductive modes are important features in the phylogenetic organization of elasmobranchs [101,102]. Aplacental viviparity, a reproductive mode in which embryos develop inside the mother’s body without the formation of a placenta, is a significant adaptation that can influence the survival and reproductive success of species [103]. These findings are corroborated by studies showing that reproductive characteristics can be used to trace evolutionary relationships between shark and ray species [78,84]. The inclusion of *M. schmitti* in the same clade as other species with aplacental viviparity suggests that this reproductive mode may have evolved once in a common ancestor, being maintained throughout evolution due to its adaptive advantages [32,42].

One of the factors limiting the phylogenetic analysis of the family Triakidae is the lack of mtDNAs of other species, mainly those from SAO. It is still challenging to differentiate species, as well as to study population structures within the same species. Therefore, the *M. schmitti* mtDNA sequenced here provides the genetic identity for an important species living in SAO. As more efficient taxonomic classification methods are developed, these species can be more easily monitored and tracked, also making it possible to implement actions to reinforce coastal inspection to prevent predatory fishing. In addition, specific species populations can be estimated to determine specific time series of catches in Brazilian waters.

Finally, the monophyletic organization of *M. schmitti* and its association with other aplacental viviparous species highlight the importance of reproductive characteristics in the phylogenetic structuring of the Triakidae [103]. These results highlight the need to integrate morphological, ecological and molecular data for a comprehensive understanding of the evolutionary trajectories of these sharks, aiding in the development of effective management and conservation strategies.

## 5. Conclusions

These results provide an understanding of the *M. schmitti* mtDNA, with complete mitochondrial genome sequence and structure. The phylogenetic analysis clarifies the evolutionary relationships within the Triakidae family, reinforcing that *Mustelus* and *Triakis* are not monophyletic. These findings are important for accurate species identification, contributing to the conservation and management of shark and other coastal species, which are under heavy pressure due to commercial fishing.

## Figures and Tables

**Table 1:** General information and nucleotide composition for the mtDNAs of shark species of the Carcharhiniform order and one outgroup.

Family	Species	Size (bp)	AT%	GenBank
Carcharhinidae:	*Carcharhinus acronotus*	16,719	61.6	NC_024055
	*Carcharhinus albimarginatus*	16,706	61.4	NC_047239
	*Carcharhinus amblyrhynchoides*	16,705	61.8	NC_023948
	*Carcharhinus amblyrhynchos*	16,705	61.6	NC_047238
	*Carcharhinus amboinensis*	16,704	62.0	NC_026696
	*Carcharhinus brachyurus*	16,704	61.7	NC_057525
	*Carcharhinus brevipinna*	16,706	61.4	NC_027081
	*Carcharhinus falciformis*	16,677	61.4	NC_042256
	*Carcharhinus leucas*	16,704	62.6	NC_023522
	*Carcharhinus limbatus*	16,705	61.7	NC_057057
	*Carcharhinus longimanus*	16,706	61.5	NC_025520
	*Carcharhinus macloti*	16,701	60.8	NC_024862
	*Carcharhinus melanopterus*	16,706	61.4	NC_024284
	*Carcharhinus obscurus*	16,706	61.5	NC_020611
	*Carcharhinus perezii*	16,709	61.5	MW528216
	*Carcharhinus plumbeus*	16,706	61.2	NC_024596
	*Carcharhinus sorrah*	16,707	61.0	NC_023521
	*Carcharhinus tjutjot*	16,705	60.6	NC_026871
	*Glyphis fowlerae*	16,704	60.6	NC_028342
	*Glyphis garricki*	16,702	60.8	NC_023361
	*Glyphis glyphis*	16,701	61.0	NC_021768
	*Lamiopsis temminckii*	16,708	61.1	NC_028341
	*Lamiopsis tephrodes*	16,705	61.2	NC_028340
	*Loxodon macrorhinus*	16,702	61.1	NC_029843
	*Prionace glauca*	16,705	62.5	NC_022819
	*Rhizoprionodon acutus*	16,693	63.0	NC_046016
	*Scoliodon laticaudus*	16,695	63.1	NC_042504
	*Scoliodon macrorhynchos*	16,693	63.1	NC_018052
	*Triaenodon obesus*	16,700	61.1	NC_026287
Galeocerdidae:	*Galeocerdo cuvier*	16,703	63.1	NC_022193
Hemigaleidae:	*Hemigaleus microstoma*	16,701	60.1	NC_029400
	*Hemipristis elongata*	16,691	63.0	NC_032065
Proscylliidae:	*Proscyllium habereri*	16,708	62.1	NC_030216
Pseudotriakidae:	*Pseudotriakis microdon*	16,700	63.6	NC_022735
Scyliorhinidae:	*Cephaloscyllium fasciatum*	16,703	61.9	MZ424309
	*Cephaloscyllium umbratile*	16,698	62.1	NC_029399
	*Galeus melastomus*	16,706	63.2	NC_049881
	*Halaelurus buergeri*	19,100	61.1	NC_031811
	*Parmaturus melanobranchus*	16,687	62.5	NC_056784
	*Poroderma pantherinum*	16,686	61.1	NC_043830
	*Scyliorhinus canicula*	16,697	62.0	NC_001950
	*Scyliorhinus torazame*	17,861	61.8	AP019520
Sphyrnidae:	*Eusphyra blochii*	16,727	61.3	NC_031812
	*Sphyrna lewini*	16,726	60.5	NC_022679
	*Sphyrna mokarran*	16,719	61.4	NC_035491
	*Sphyrna tiburo*	16,723	60.7	NC_028508
	*Sphyrna zygaena*	16,731	61.7	NC_025778
Triakidae:	*Galeorhinus galeus*	17,488	62.0	ON652874
	*Hemitriakis japanica*	17,301	60.0	KJ617039
	*Mustelus asterias* *	16,708	61.5	ON652873
	*Mustelus canis*	16,758	60.8	OP056805
	*Mustelus griseus* *	16,754	61.0	NC_023527
	*Mustelus manazo* *	16,707	61.8	NC_000890
	*Mustelus mosis*	16,755	60.7	ON075077
	*Mustelus mustelus* *	16,755	60.8	NC_039629
	*Mustelus norrisi*	16,769	61.2	OP056963
	*Mustelus palumbes* *	16,708	61.5	NC_077463
	*Mustelus schmitti* **	16,764	61.4	PQ182775
	*Triakis megalopterus*	16,746	61.3	ON075075
	*Triakis semifasciata*	16,613	61.2	NC_077588
Hemiscylliidae	*Chiloscyllium griseum*	16,755	63.9	NC_017882
Lamnidae	*Lamna ditropis*	16,702	61.1	NC_024269

* Reference mtDNA complete genomes used for assembling the *M. schmitti*; ** obtained in this study.

**Table 2:** Annotation of the complete mtDNAs of *Mustelus schmitti*.

Name	Codon Start	Codon Stop	Anti-Codon	AT%	CG%	Type	Position from	Position to	Length	Strand
*D-loop*				63.9	35.1	*D-loop*	15641	16763	1123	H
tRNA-*Pro*			TGG	43.5	56.5	tRNA	15572	15640	69	L
tRNA-*Thr*			TGT	57.0	43.0	tRNA	15498	15569	72	H
*Cytb*	ATG	TAG		59.3	40.7	gene	14353	15497	1145	H
tRNA-*Glu*			TTC	68.6	31.4	tRNA	14281	14350	70	L
*ND6*	CTA	CAT		61.5	38.1	gene	13759	14280	522	L
*ND5*	ATG	TAA		63.4	36.5	gene	11934	13763	1830	H
tRNA-*Leu2*			TAG	59.7	40.2	tRNA	11862	11933	72	H
tRNA-*Ser2*			GCT	49.3	50.8	tRNA	11795	11861	67	H
tRNA-*His*			GTG	79.7	20.3	tRNA	11726	11794	69	H
*ND4*	ATG	T-		62.4	37.6	gene	10345	11725	1381	H
*ND4L*	ATG	TAA		58.3	41.7	gene	10055	10351	297	H
tRNA-*Arg*			TCG	67.1	32.9	tRNA	9985	10054	70	H
*ND3*	ATG	TAG		56.4	43.5	gene	9636	9984	349	H
tRNA-*Gly*			TCC	70.0	30.0	tRNA	9566	9635	70	H
*COXIII*	ATG	TAA		57.9	42.1	gene	8778	9563	786	H
*ATPase6*	ATG	TAA		64.2	35.7	gene	8095	8777	683	H
*ATPase8*	ATG	TAA		72.0	27.9	gene	7937	8104	168	H
tRNA-*Lys*			TTT	60.8	39.2	tRNA	7862	7935	74	H
*COXII*	ATG	T-		61.8	38.2	gene	7171	7861	691	H
tRNA-*Asp*			GTC	62.9	37.2	tRNA	7094	7163	70	H
tRNA-*Ser1*			TGA	53.5	46.5	tRNA	7020	7090	71	L
*COXI*	GTG	TAA		61.5	38.5	gene	5462	7018	1557	H
tRNA-*Tyr*			GTA	47.2	50.0	tRNA	5391	5460	70	L
tRNA-*Cys*			GCA	49.2	50.7	tRNA	5321	5389	69	L
tRNA-*Asn*			GTT	61.6	38.4	tRNA	5213	5285	73	L
tRNA-*Ala*			TGC	66.6	33.3	tRNA	5144	5212	69	L
tRNA-*Trp*			CCA	67.6	32.4	tRNA	5072	5142	71	H
*ND2*	ATG	TAG		62.2	37.8	gene	4027	5071	1045	H
tRNA-*Met*			CAT	57.9	42.0	tRNA	3958	4026	69	H
tRNA-*Gln*			TTG	65.3	34.7	tRNA	3886	3957	72	L
tRNA-*Ile*			GAT	54.3	45.7	tRNA	3815	3884	70	H
*ND1*	ATG	TAA		60.3	39.7	gene	2840	3814	975	H
tRNA-*Leu1*			TAA	54.7	45.3	tRNA	2765	2839	75	H
16S rRNA				62.5	37.4	rRNA	1095	2764	1670	H
tRNA-*Val*			TAV	56.9	43.0	tRNA	1023	1094	72	H
12S rRNA				58.2	41.8	rRNA	70	1022	953	H
tRNA-*Phe*			GAA	60.9	39.1	tRNA	1	69	69	H

## Data Availability

Data are contained within the article.

## References

[1] Compagno L.J. (1977). Phyletic relationships of living sharks and rays. Am. Zool..

[2] Stein R.W., Mull C.G., Kuhn T.S., Aschliman N.C., Davidson L.N., Joy J.B., Smith G.J., Dulvy N.K., Mooers A.O. (2018). Global priorities for conserving the evolutionary history of sharks, rays and chimaeras. Nat. Ecol. Evol..

[3] Ebert D.A., Dando M., Fowler S. (2021). Sharks of the World: A Complete Guide.

[4] Weigmann S. (2016). Annotated checklist of the living sharks, batoids and chimaeras (Chondrichthyes) of the world, with a focus on biogeographical diversity. J. Fish Biol..

[5] Compagno L. (1984). Pt. 2: Carcharhiniformes. Sharks of the World. An Annotated and Illustrated Catalogue of Shark Species Known to Date.

[6] Rosa M.R., Gadig O.B.F. (2010). Taxonomic comments and an identification key to species for the smooth-hound sharks genus *Mustelus* Link, 1790 (Chondrichthyes: Triakidae) from the western South Atlantic. Pan-Am. J. Aquat. Sci..

[7] BRASIL Portaria MMA Nº 148, de 07 de junho de 2022. Lista Nacional Oficial de Espécies da Fauna Ameaçadas de Extinção. Anexo I e II. Portaria MMA Nº 148, de 07 de Junho de 2022 2022a, Portaria MMA Nº 148/2022, 116. https://www.icmbio.gov.br/cepsul/images/stories/legislacao/Portaria/2020/P_mma_148_2022_altera_anexos_P_mma_443_444_445_2014_atualiza_especies_ameacadas_extincao.pdf.

[8] BRASIL Portaria MMA Nº 300, de 13 de dezembro de 2022. Lista Nacional Oficial de Espécies da Fauna Ameaçadas de Extinção. Anexo I e II. Portaria MMA Nº 300, de 13 de Dezembro de 2022 2022b, Portaria MMA Nº 300/2022, 90. https://www.icmbio.gov.br/cepsul/images/stories/legislacao/Portaria/2022/P_gm_mma_300_2022_reconhece_lista_nacional_spp_ameacadas_extincao.pdf.

[9] Nisa-Castro-Neto W. (2001). Análise de Pesca de Carcharias Taurus Rafinesque, 1810 (Chondrichthyes, Odontaspididae) e Seu Declínio Nas Regiões Sul e Sudeste do Brasil.

[10] Santos P.R.S., Balanin S., Gadig O.B.F., Garrone-Neto D. (2022). The historical and contemporary knowledge on the elasmobranchs of Cananeia and adjacent waters, a coastal marine hotspot of southeastern Brazil. Reg. Stud. Mar. Sci..

[11] Dulvy N.K., Pacoureau N., Rigby C.L., Pollom R.A., Jabado R.W., Ebert D.A., Finucci B., Pollock C.M., Cheok J., Derrick D.H. (2021). Overfishing drives over one-third of all sharks and rays toward a global extinction crisis. Curr. Biol..

[12] Instituto Chico Mendes de Conservação da Biodiversidade (2018). Livro Vermelho da Fauna Brasileira Ameaçada de Extinção.

[13] Pollom R., Barreto R., Charvet P., Chiaramonte G.E., Cuevas J.M., Herman K., Montealegre-Quijano S., Motta F., Paesch L., Rincon G. (2020). Mustelus fasciatus. The IUCN Red List of Threatened Species 2020.

[14] Pollom R., Barreto R., Charvet P., Chiaramonte G.E., Cuevas J.M., Herman K., Montealegre-Quijano S., Motta F., Paesch L., Rincon G. (2020). Mustelus schmitti. The IUCN Red List of Threatened Species 2020.

[15] Essington T.E., Moriarty P.E., Froehlich H.E., Hodgson E.E., Koehn L.E., Oken K.L., Siple M.C., Stawitz C.C. (2015). Fishing amplifies forage fish population collapses. Proc. Natl. Acad. Sci. USA.

[16] Queiroz N., Humphries N.E., Mucientes G., Hammerschlag N., Lima F.P., Scales K.L., Miller P.I., Sousa L.L., Seabra R., Sims D.W. (2016). Ocean-wide tracking of pelagic sharks reveals extent of overlap with longline fishing hotspots. Proc. Natl. Acad. Sci. USA.

[17] Dario F.D., Alves C.B., Boos H., Frédou F.L., Lessa R.P., Mincarone M.M., Pinheiro M.A., Polaz C.N., Reis R.E., Rocha L.A. (2015). A better way forward for Brazil’s fisheries. Science.

[18] Camacho-Oliveira R.B., Daneluz C.M., do Prado F.D., Utsunomia R., Rodrigues Jr C.E., Foresti F., Porto-Foresti F. (2020). DNA barcode reveals the illegal trade of rays commercialized in fishmongers in Brazil. Forensic Sci. Int. Synerg..

[19] Bunholi I.V., da Silva Ferrette B.L., De Biasi J.B., de Oliveira Magalhães C., Rotundo M.M., Oliveira C., Foresti F., Mendonça F.F. (2018). The fishing and illegal trade of the angelshark: DNA barcoding against misleading identifications. Fish. Res..

[20] Dudgeon C., Blower D., Broderick D., Giles J., Holmes B., Kashiwagi T., Krueuck N., Morgan J., Tillett B., Ovenden J. (2012). A review of the application of molecular genetics for fisheries management and conservation of sharks and rays. J. Fish Biol..

[21] Pereyra S., Garcia G., Miller P., Oviedo S., Domingo A. (2010). Low genetic diversity and population structure of the narrownose shark (*Mustelus schmitti*). Fish. Res..

[22] Miranda L.d., Vooren C. (2003). Captura e esforço da pesca de elasmobrânquios demersais no sul do Brasil nos anos de 1975 a 1997. Frente Marítimo.

[23] Vooren C., Oddone M. (2019). The diversity of the chondrichthyans of the far south of Brazil: The species, their origins, and their reproductive modes. Ciencias Marino-Costeras en el Umbral del Siglo XXI. Desafios em Latinoamérica y el Caribe.

[24] Massa A., Hozbor N., Lucifora L., Colonello J. (2003). Sugerencias de capturas para el año 2003 de gatuzo (*Mustelus* spp.), peces angel (*Squatina* spp.) y rayas costeras. Informe Técnico Interno INIDEP.

[25] Oddone M., Paesch L., Norbis W. (2005). Reproductive biology and seasonal distribution of the patagonian smoothhound *Mustelus schmitti* (Elasmobranchii: Triakidae) in the Rio de La Plata oceanic front, South-Western Atlantic. J. Mar. Biol. Assoc. U. K..

[26] Vooren C., Klippel S., Galina A. (2005). Os elasmobrânquios das águas costeiras da Plataforma Sul. Ações Para a Conservação de Tubarões e Raias No Sul do Brasil.

[27] Pereyra I., Orlando L., Norbis W., Paesch L. (2008). Spatial and temporal variation of length and sex composition of the narrownose smooth-hound *Mustelus schmitti* Springer, 1939 in the trawl fishery off the oceanic coast of Uruguay during 2004. Rev. Biol. Mar. Oceanogr..

[28] Chiaramonte G.E., Pettovello A.D. (2000). The biology of *Mustelus schmitti* in southern Patagonia, Argentina. J. Fish Biol..

[29] Begg G.A., Friedland K.D., Pearce J.B. (1999). Stock identification and its role in stock assessment and fisheries management: An overview. Fish. Res..

[30] Pimiento C., Albouy C., Silvestro D., Mouton T.L., Velez L., Mouillot D., Judah A.B., Griffin J.N., Leprieur F. (2023). Functional diversity of sharks and rays is highly vulnerable and supported by unique species and locations worldwide. Nat. Commun..

[31] Reiss H., Hoarau G., Dickey-Collas M., Wolff W.J. (2009). Genetic population structure of marine fish: Mismatch between biological and fisheries management units. Fish Fish..

[32] López J.A., Ryburn J.A., Fedrigo O., Naylor G.J. (2006). Phylogeny of sharks of the family Triakidae (Carcharhiniformes) and its implications for the evolution of carcharhiniform placental viviparity. Mol. Phylogenetics Evol..

[33] Cortés F. (2007). Sustentabilidad de la Explotación del Gatuzo Mustelus schmitti, en el Ecosistema Costero Bonaerense (34–42 S). Bachelor Thesis.

[34] Lim D.D., Motta P., Mara K., Martin A.P. (2010). Phylogeny of hammerhead sharks (Family Sphyrnidae) inferred from mitochondrial and nuclear genes. Mol. Phylogenetics Evol..

[35] Dosay-Akbulut M. (2008). The phylogenetic relationship within the genus *Carcharhinus*. Comptes Rendus Biol..

[36] Cunha D.B., Silva Rodrigues-Filho L.F., Luna Sales J.B. (2017). A Review of the Mitogenomic Phylogeny of the Chondrichthyes. Chondrichthyes-Multidisciplinary Approach.

[37] Naylor G.J., Ryburn J., Fedrigo O., Lopez J. (2005). Phylogenetic relationships among the major lineages of modern elasmobranchs. Reprod. Biol. Phylogeny.

[38] Maisey J., Naylor G., Ward D., Arratia G., Tintori A. (2004). Mesozoic elasmobranchs, neoselachian phylogeny and the rise of modern elasmobranch diversity. Mesoz. Fishes 3-Syst. Paleoenviron. Biodivers..

[39] McEachran J.D., Aschliman N. (2004). Phylogeny of Batoidea.

[40] Renz A.J., Meyer A., Kuraku S. (2013). Revealing less derived nature of cartilaginous fish genomes with their evolutionary time scale inferred with nuclear genes. PLoS ONE.

[41] Kousteni V., Mazzoleni S., Vasileiadou K., Rovatsos M. (2021). Complete mitochondrial DNA genome of nine species of sharks and rays and their phylogenetic placement among modern elasmobranchs. Genes.

[42] Iglésias S.P., Lecointre G., Sellos D.Y. (2005). Extensive paraphylies within sharks of the order Carcharhiniformes inferred from nuclear and mitochondrial genes. Mol. Phylogenetics Evol..

[43] Kiser H., Skufca K., Bemis K.E., Baeza J.A. (2024). Comparative analysis of the mitochondrial genomes of Smoothhound sharks provide insight into the phylogenetic relationships within the family Triakidae. Gene Rep..

[44] Winn J.C., Maduna S.N., Bester-van der Merwe A.E. (2024). A comprehensive phylogenomic study unveils evolutionary patterns and challenges in the mitochondrial genomes of Carcharhiniformes: A focus on Triakidae. Genomics.

[45] Wang C., Lai T., Ye P., Yan Y., Feutry P., He B., Huang Z., Zhu T., Wang J., Chen X. (2022). Novel duplication remnant in the first complete mitogenome of *Hemitriakis japanica* and the unique phylogenetic position of family Triakidae. Gene.

[46] Heinicke M., Naylor G., Hedges S. (2009). Cartilaginous fishes (Chondrichthyes). Timetree Life.

[47] Maduna S.N., Rossouw C., Da Silva C., Soekoe M., Bester-van der Merwe A.E. (2017). Species identification and comparative population genetics of four coastal houndsharks based on novel NGS-mined microsatellites. Ecol. Evol..

[48] Nickum J., Bart H., Bowser P., Greer I., Hubbs C., Jenkins J., MacMillan J., Rachlin J., Rose J., Sorensen P. (2004). Guidelines for the use of fishes in research. Am. Fish. Soc..

[49] Bolger A.M., Lohse M., Usadel B. (2014). Trimmomatic: A flexible trimmer for Illumina sequence data. Bioinformatics.

[50] Bankevich A., Nurk S., Antipov D., Gurevich A.A., Dvorkin M., Kulikov A.S., Lesin V.M., Nikolenko S.I., Pham S., Prjibelski A.D. (2012). SPAdes: A new genome assembly algorithm and its applications to single-cell sequencing. J. Comput. Biol..

[51] Mikheenko A., Valin G., Prjibelski A., Saveliev V., Gurevich A. (2016). Icarus: Visualizer for de novo assembly evaluation. Bioinformatics.

[52] Iwasaki W., Fukunaga T., Isagozawa R., Yamada K., Maeda Y., Satoh T.P., Sado T., Mabuchi K., Takeshima H., Miya M. (2013). MitoFish and MitoAnnotator: A mitochondrial genome database of fish with an accurate and automatic annotation pipeline. Mol. Biol. Evol..

[53] Lagesen K., Hallin P., Rødland E.A., Stærfeldt H.-H., Rognes T., Ussery D.W. (2007). RNAmmer: Consistent and rapid annotation of ribosomal RNA genes. Nucleic Acids Res..

[54] Chan P.P., Lowe T.M. (2019). TRNAscan-SE: Searching for TRNA Genes in Genomic Sequences.

[55] Tamura K., Stecher G., Kumar S. (2021). MEGA11: Molecular Evolutionary Genetics Analysis Version 11. Mol. Biol. Evol..

[56] Griffin A.M., Griffin H.G., Higgins D.G. (1994). CLUSTAL V: Multiple alignment of DNA and protein sequences. Comput. Anal. Seq. Data Part II.

[57] Trifinopoulos J., Nguyen L.-T., von Haeseler A., Minh B.Q. (2016). W-IQ-TREE: A fast online phylogenetic tool for maximum likelihood analysis. Nucleic Acids Res..

[58] Kalyaanamoorthy S., Minh B.Q., Wong T.K., Von Haeseler A., Jermiin L.S. (2017). ModelFinder: Fast model selection for accurate phylogenetic estimates. Nat. Methods.

[59] Hoang D.T., Chernomor O., Von Haeseler A., Minh B.Q., Vinh L.S. (2018). UFBoot2: Improving the ultrafast bootstrap approximation. Mol. Biol. Evol..

[60] Alvarenga M., Bunholi I.V., de Brito G.R., Siqueira M.V.B.M., Domingues R.R., Charvet P., Foresti F., Solé-Cava A.M., da Cruz V.P. (2024). Fifteen years of elasmobranchs trade unveiled by DNA tools: Lessons for enhanced monitoring and conservation actions. Biol. Conserv..

[61] Kenchington E.L. (2003). The Effects of Fishing on Species and Genetic Diversity. Responsible Fish. Mar. Ecosyst..

[62] Villate-Moreno M., Cubillos-M J.C., Stibor H., Crawford A.J., Straube N. (2022). Molecular identification and first demographic insights of sharks based on artisanal fisheries bycatch in the Pacific Coast of Colombia: Implications for conservation. PeerJ.

[63] Douady C.J., Dosay M., Shivji M.S., Stanhope M.J. (2003). Molecular phylogenetic evidence refuting the hypothesis of Batoidea (rays and skates) as derived sharks. Mol. Phylogenetics Evol..

[64] Martin A. (2001). The phylogenetic placement of Chondrichthyes: Inferences from analysis of multiple genes and implications for comparative studies. Genetica.

[65] Kamal S.A., Baeza J.A. (2024). Detailed characterization of the complete mitochondrial genome of the oceanic whitetip shark *Carcharhinus longimanus* (Poey, 1861). Mol. Biol. Rep..

[66] Inoue J.G., Miya M., Lam K., Tay B.-H., Danks J.A., Bell J., Walker T.I., Venkatesh B. (2010). Evolutionary origin and phylogeny of the modern holocephalans (Chondrichthyes: Chimaeriformes): A mitogenomic perspective. Mol. Biol. Evol..

[67] Zhu K.-C., Liang Y.-Y., Wu N., Guo H.-Y., Zhang N., Jiang S.-G., Zhang D.-C. (2017). Sequencing and characterization of the complete mitochondrial genome of Japanese Swellshark (*Cephalloscyllium umbratile*). Sci. Rep..

[68] Palacios-Barreto P., Mar-Silva A.F., Bayona-Vasquez N.J., Adams D.H., Díaz-Jaimes P. (2023). Characterization of the complete mitochondrial genome of the Brazilian cownose ray *Rhinoptera brasiliensis* (Myliobatiformes, Rhinopteridae) in the western Atlantic and its phylogenetic implications. Mol. Biol. Rep..

[69] Miya M., Takeshima H., Endo H., Ishiguro N.B., Inoue J.G., Mukai T., Satoh T.P., Yamaguchi M., Kawaguchi A., Mabuchi K. (2003). Major patterns of higher teleostean phylogenies: A new perspective based on 100 complete mitochondrial DNA sequences. Mol. Phylogenetics Evol..

[70] Condamine F.L., Romieu J., Guinot G. (2019). Climate cooling and clade competition likely drove the decline of lamniform sharks. Proc. Natl. Acad. Sci. USA.

[71] Klein J.D., Maduna S.N., Dicken M.L., da Silva C., Soekoe M., McCord M.E., Potts W.M., Hagen S.B., Bester-van der Merwe A.E. (2024). Local adaptation with gene flow in a highly dispersive shark. Evol. Appl..

[72] Sabadin D.E., Lucifora L.O., Barbini S.A., Figueroa D.E., Kittlein M. (2020). Towards regionalization of the chondrichthyan fauna of the Southwest Atlantic: A spatial framework for conservation planning. ICES J. Mar. Sci..

[73] Kraft D.W., Conklin E.E., Barba E.W., Hutchinson M., Toonen R.J., Forsman Z.H., Bowen B.W. (2020). Genomics versus mtDNA for resolving stock structure in the silky shark (*Carcharhinus falciformis*). PeerJ.

[74] Domingues R.R., Hilsdorf A.W.S., Gadig O.B.F. (2018). The importance of considering genetic diversity in shark and ray conservation policies. Conserv. Genet..

[75] Hara Y., Yamaguchi K., Onimaru K., Kadota M., Koyanagi M., Keeley S.D., Tatsumi K., Tanaka K., Motone F., Kageyama Y. (2018). Shark genomes provide insights into elasmobranch evolution and the origin of vertebrates. Nat. Ecol. Evol..

[76] Read T.D., Petit R.A., Joseph S.J., Alam M.T., Weil M.R., Ahmad M., Bhimani R., Vuong J.S., Haase C.P., Webb D.H. (2017). Draft sequencing and assembly of the genome of the world’s largest fish, the whale shark: *Rhincodon typus* Smith 1828. BMC Genom..

[77] Kuraku S. (2021). Shark and ray genomics for disentangling their morphological diversity and vertebrate evolution. Dev. Biol..

[78] Ho S.Y., Lanfear R., Bromham L., Phillips M.J., Soubrier J., Rodrigo A.G., Cooper A. (2011). Time-dependent rates of molecular evolution. Mol. Ecol..

[79] Díaz-Jaimes P., Bayona-Vásquez N.J., Adams D.H., Uribe-Alcocer M. (2016). Complete mitochondrial DNA genome of bonnethead shark, *Sphyrna tiburo*, and phylogenetic relationships among main superorders of modern elasmobranchs. Meta Gene.

[80] Heist E.J. (2012). Genetics of sharks, skates and rays. Biology of Sharks and Their Relatives.

[81] Stingo V., Capriglione T., Rocco L., Improta R., Morescalchi A. (1989). Genome size and AT rich DNA in selachians. Genetica.

[82] Huang X., Yu J., Chen H., Chen X., Wang J. (2016). Complete mitochondrial genome and the phylogenetic position of the snaggletooth shark *Hemipristis elongata* (Carcharhiniformes: Hemigaleidae). Mitochondrial DNA Part B.

[83] Brée B., Condamine F.L., Guinot G. (2022). Combining palaeontological and neontological data shows a delayed diversification burst of carcharhiniform sharks likely mediated by environmental change. Sci. Rep..

[84] Dudgeon C.L., Corrigan S., Yang L., Allen G.R., Erdmann M.V., Sugeha H.Y., White W.T., Naylor G.J. (2020). Walking, swimming or hitching a ride? Phylogenetics and biogeography of the walking shark genus *Hemiscyllium*. Mar. Freshw. Res..

[85] Sayyari E., Mirarab S. (2018). Testing for polytomies in phylogenetic species trees using quartet frequencies. Genes.

[86] McLay T.G., Fowler R.M., Fahey P.S., Murphy D.J., Udovicic F., Cantrill D.J., Bayly M.J. (2023). Phylogenomics reveals extreme gene tree discordance in a lineage of dominant trees: Hybridization, introgression, and incomplete lineage sorting blur deep evolutionary relationships despite clear species groupings in Eucalyptus subgenus Eudesmia. Mol. Phylogenetics Evol..

[87] Jeiter J., Smets E. (2023). Integrating comparative morphology and development into evolutionary research. Taxon.

[88] Lücking R., Leavitt S.D., Hawksworth D.L. (2021). Species in lichen-forming fungi: Balancing between conceptual and practical considerations, and between phenotype and phylogenomics. Fungal Divers..

[89] Steenwyk J.L., Li Y., Zhou X., Shen X.-X., Rokas A. (2023). Incongruence in the phylogenomics era. Nat. Rev. Genet..

[90] DeSalle R., Goldstein P. (2019). Review and interpretation of trends in DNA barcoding. Front. Ecol. Evol..

[91] Guo M., Yuan C., Tao L., Cai Y., Zhang W. (2022). Life barcoded by DNA barcodes. Conserv. Genet. Resour..

[92] Human B.A., Owen E.P., Compagno L.J., Harley E.H. (2006). Testing morphologically based phylogenetic theories within the cartilaginous fishes with molecular data, with special reference to the catshark family (Chondrichthyes; Scyliorhinidae) and the interrelationships within them. Mol. Phylogenetics Evol..

[93] Corrigan S., Beheregaray L.B. (2009). A recent shark radiation: Molecular phylogeny, biogeography and speciation of wobbegong sharks (family: Orectolobidae). Mol. Phylogenetics Evol..

[94] Compagno L.J. (1999). Systematics and body form. Sharks, Skates and Rays: The Biology of Elasmobranch Fishes.

[95] Rasmussen A.S., Arnason U. (1999). Phylogenetic studies of complete mitochondrial DNA molecules place cartilaginous fishes within the tree of bony fishes. J. Mol. Evol..

[96] Boomer J.J., Harcourt R.G., Francis M.P., Stow A.J. (2012). Genetic divergence, speciation and biogeography of *Mustelus* (sharks) in the central Indo-Pacific and Australasia. Mol. Phylogenetics Evol..

[97] Maduna S.N. (2017). Unravelling the Mystery of the Shark Genus Mustelus in Southern Africa using a Multidisciplinary Approach. Ph.D. Thesis.

[98] Sandoval-Castillo J., Beheregaray L.B. (2020). Oceanographic heterogeneity influences an ecological radiation in elasmobranchs. J. Biogeogr..

[99] Da Silva Rodrigues-Filho L.F., da Costa Nogueira P., Sodré D., da Silva Leal J.R., Nunes J.L.S., Rincon G., Lessa R.P.T., Sampaio I., Vallinoto M., Ready J.S. (2023). Evolutionary history and taxonomic reclassification of the critically endangered daggernose shark, a species endemic to the Western Atlantic. J. Zool. Syst. Evol. Res..

[100] Sternes P.C., Schmitz L., Higham T.E. (2024). The rise of pelagic sharks and adaptive evolution of pectoral fin morphology during the Cretaceous. Curr. Biol..

[101] Clarke C.R., Karl S.A., Horn R.L., Bernard A.M., Lea J.S., Hazin F.H., Prodöhl P.A., Shivji M.S. (2015). Global mitochondrial DNA phylogeography and population structure of the silky shark, *Carcharhinus falciformis*. Mar. Biol..

[102] Karl S., Castro A., Lopez J., Charvet P., Burgess G. (2011). Phylogeography and conservation of the bull shark (*Carcharhinus leucas*) inferred from mitochondrial and microsatellite DNA. Conserv. Genet..

[103] Harnlett W.C. (2011). Placentatrophy in Sharks. Reproductive Biology and Phylogeny of Chondrichthyes.

